# Layer separation mapping and consolidation evaluation of a fifteenth century panel painting using terahertz time-domain imaging

**DOI:** 10.1038/s41598-022-25013-8

**Published:** 2022-12-05

**Authors:** Frances E. M. Lambert, Jan Ornik, Naja-Anissa Staats, Alexander Jäckel, Goretti G. Hernandez-Cardoso, Jochen Taiber, Eva-Maria Stübling, Benjamin Rudolph, Oliver Mack, Hans Portsteffen, Enrique Castro-Camus, Martin Koch

**Affiliations:** 1 Department of Physics and Material Sciences Center, Philipps-Universität Marburg, Renthof 5, 35032 Marburg, Germany; 2 Cologne Institute of Conservation Sciences, Technische Hochschule Köln, Ubierring 40, Cologne, 50678 Germany; 3 Germanisches Nationalmuseum, Kartäusergasse 1, Nuremberg, 90402 Germany

**Keywords:** Imaging and sensing, Terahertz optics

## Abstract

Over time, artworks often sustain paint layer separation and air gaps within their internal structure due to storage conditions and past restoration efforts. Because of this, paint layer consolidation interventions are an essential activity for art conservators. However, it is difficult to determine the exact location and the extent of layer separation on a piece of art in a non-invasive way, and even more difficult to evaluate the success of a consolidation intervention. In this work, a fifteenth-century wood panel painting was analyzed using terahertz time-domain imaging before and after it was consolidated. Using the terahertz data, it was possible to determine the areas on the artwork in need of consolidation and aid the intervention. The analysis of the after data allowed for the control and determination of the success of the consolidation effort in a non-destructive manner.

## Introduction

One of the most common factors that affect paintings over time is the detachment of paint layers due to age, environmental fluctuations, and storage conditions. The specific conservation process used to address this problem is known as consolidation, and is an essential activity for art conservators. Paint layer detachment can manifest itself in the form of lifting or subsurface air voids and gaps^1^. Consolidation processes present many difficulties for conservators as it is challenging to determine the exact regions in need of consolidation and identify the type of detachment or layer irregularity present. Furthermore, it is even more difficult to evaluate the success of a consolidation effort after it has been done. Because of this, many artworks undergo multiple consolidation efforts to repair regions that have repeatedly become detached.

Over the past years, different technologies such as micro-indentation^2^, Raman spectroscopy^3^, spectrophotometry^4^ have been used to monitor and control consolidation interventions. However, most of the methods used provide information regarding the chemical composition and distribution of consolidation agents rather than the internal structure of the artwork. Furthermore, these methods have been more widely used to monitor consolidation efforts on wall paintings instead of easel and panel paintings. Other technologies such as optical computed tomography (OCT) have been used as a diagnostic tool to provide information regarding the internal structure of heritage objects^5^. OCT has even been used to monitor consolidation efforts on glass paintings^6^. However, this technique is limited by its permeability of paint layers. Therefore, the available techniques that are practical for monitoring consolidation interventions are limited. More recently, terahertz (THz) time-domain imaging (TDI) has been used for this^1^.

Terahertz corresponds to the frequency band located on the electromagnetic spectrum between the infrared and the microwave range (0.1–10 THz). Scientists were first able to generate radiation in this range through photoexcitation of carriers in semiconducting materials using ultrashort laser pulses, which eventually led to the development of what is now known as terahertz time-domain spectroscopy (THz-TDS)^7^. Terahertz systems are now commercially available, and applications for this type of spectroscopy have been found in medicine^8^, communications^9^, art conservation^10^, industry^11^ among many others.

In the last twenty years, THz-TDS and THz-TDI have been used for the non-invasive analysis of pigments^12^, paintings^13,14^, murals^15,16^, manuscripts^17,18^, sculptures^19^, mummies^20^, metal artifacts^21^ and architectural elements^22,23^. Due to its unique characteristics, terahertz radiation, which is non-ionizing, can be transmitted through a wide range of optically opaque materials such as paper, fabric, paint, wood, ceramics, plastics, etc.^24^, which makes it a particularly attractive tool for the analysis of cultural heritage objects. THz-TDI can provide information both about the outside and inside of a sample, and has been used to reconstruct cross-sectional images of the internal structure of objects^25,26^. These stratigraphic images, also known as B-scans, are particularly useful for identifying sub-surface air gaps^19^, cracks^27^ and paint layer separation on artwork^28^, as well as monitoring the consolidation process of a painting^1^.

**Figure 1:**
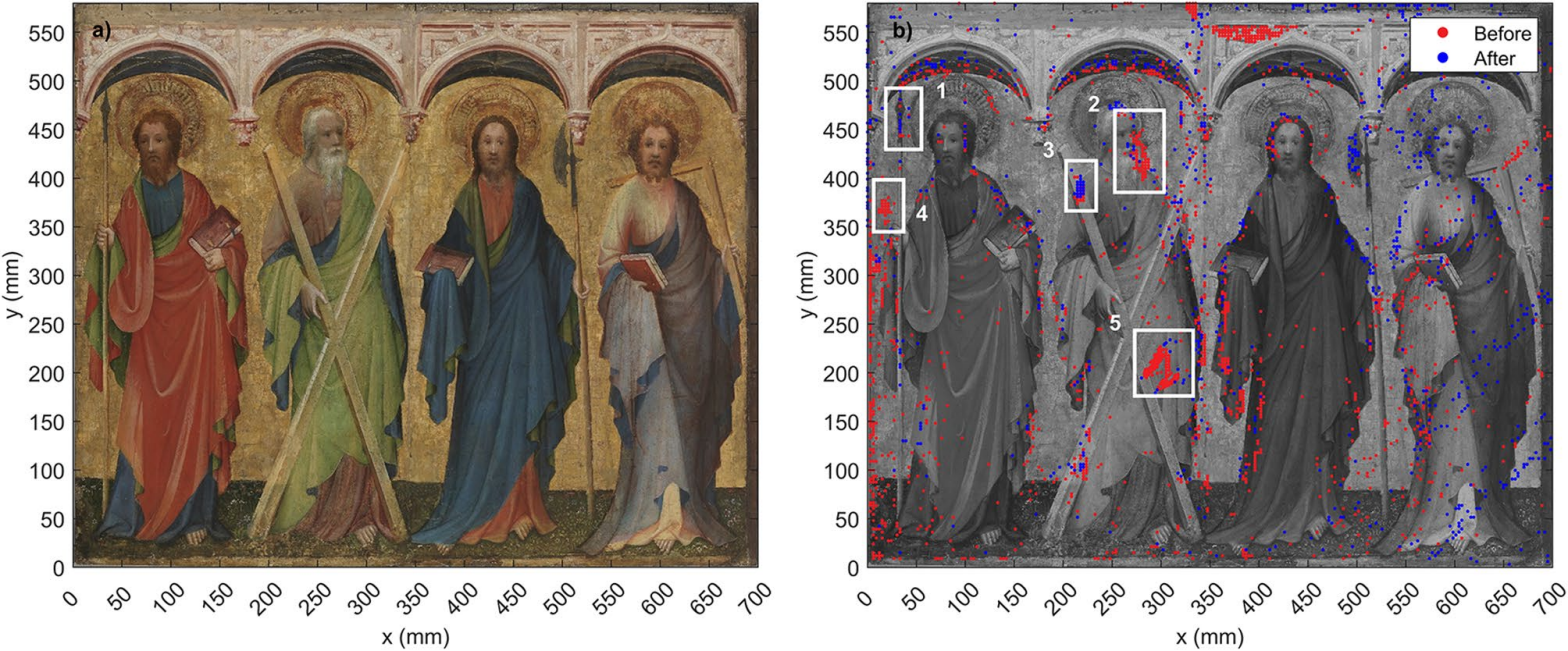
(**a**) Visible color photograph of *The Apostles Philip, Andrew, Mattias and Thomas* (c.1410-25). (**b**) THz mapping of delamination on the painting before (red) and after (blue) the consolidation effort. Delamination is denoted by dense groups of markers. Enclosed in white boxes are the five regions of interest that were selected for further analysis.

In this investigation, the object of study was a panel painting entitled *The Apostles Philip, Andrew, Mattias and Thomas* (c. 1410-25, Inv. no.: Gm 5) currently located at the Germanisches National Museum in Nuremberg, Germany. The artwork is attributed to an artist with the notational name Master of St. Laurenz, and is on permanent loan from the Bayerische Staatsgemäldesammlung/Wittelsbacher Ausgleichsfond (WAF 455) in Munich. The artwork is a 575 mm × 695 mm × 15 mm retable fragment that consists of an oak panel with a fabric support underneath the paint layers. The background and nimbs of the saints are covered with gold leaf. Figure 1a shows a visible photograph of the painting. The apostles, from left to right, are Philip, Andrew, Mattias and Thomas.

The painting has undergone several conservation efforts using different consolidant agents with varying success before the most recent intervention in 2020. Several regions on the painting have repeatedly become detached from the fabric support, as well as forming air gaps in between the layers. Before the most recent consolidation, conservators were able to identify various types of irregularities on the artwork such as paint layer lifting, delamination and air voids. The aim of this project was to determine if THz-TDI could be used to identify areas in need of consolidation on the artwork and evaluate the success of the 2020 intervention after it was done.

## Results

### THz analysis

The entire painting was scanned using THz-TDI before and after the consolidation intervention of 2020 in order to obtain two sets of data. The painting was divided into seven sections, which were each scanned with a 3 mm step size. For each data set, over 48,000 measurement points were acquired within a time frame of 50 h. One specific region that had been identified by the conservators to be particularly problematic was imaged with a step size of 1 mm. This region of interest is located on Andrew’s cross, shown in Fig. 1b as the region of interest number 5. The two data sets (before and after) were processed in order to obtain the figures shown in this section.

Through individual analysis of B-scans from regions of interest on the painting, we developed an algorithm that identified and saved the coordinates of places on the artwork where layer detachment or other irregularities were present. The coordinates were then plotted on top of a visible photograph of the painting, as shown in Fig. 1b. The red dots on the image correspond to the before intervention data, while blue dots correspond to the after data. Places where there are dense groups of markers indicate delamination, and are enclosed in white rectangles. It should be noted that the major regions of interest are located on the left half of the panel. Due to complications during the measurements, the data acquired before the intervention for the right side was unreliable. The painting is slightly warped on the right side, and it was not possible to maintain normal incidence between the THz emitter and the surface during the first measurements. This was compensated for in the after measurements, so regions on the right side of the painting where there are dense groups of blue dots indicate delamination that was still present after the consolidation effort. However, it was not possible to compare these regions with the before data.

Five regions of interest (ROIs) were selected for further analysis, and are numbered from 1 to 5. Regions 2, 4 and 5 are of particular interest, as they were also identified by the conservators in their independent analysis of the artwork previous to the THz analysis. The conservators determined that according to the type of separation present in these three regions, they were in need of consolidation. Regions 1 and 3 were not identified by conservators, but were identified by the algorithm using the THz data. These two regions have dense red and blue dots, indicating layer separation present in both the before and after data.

**Figure 2:**
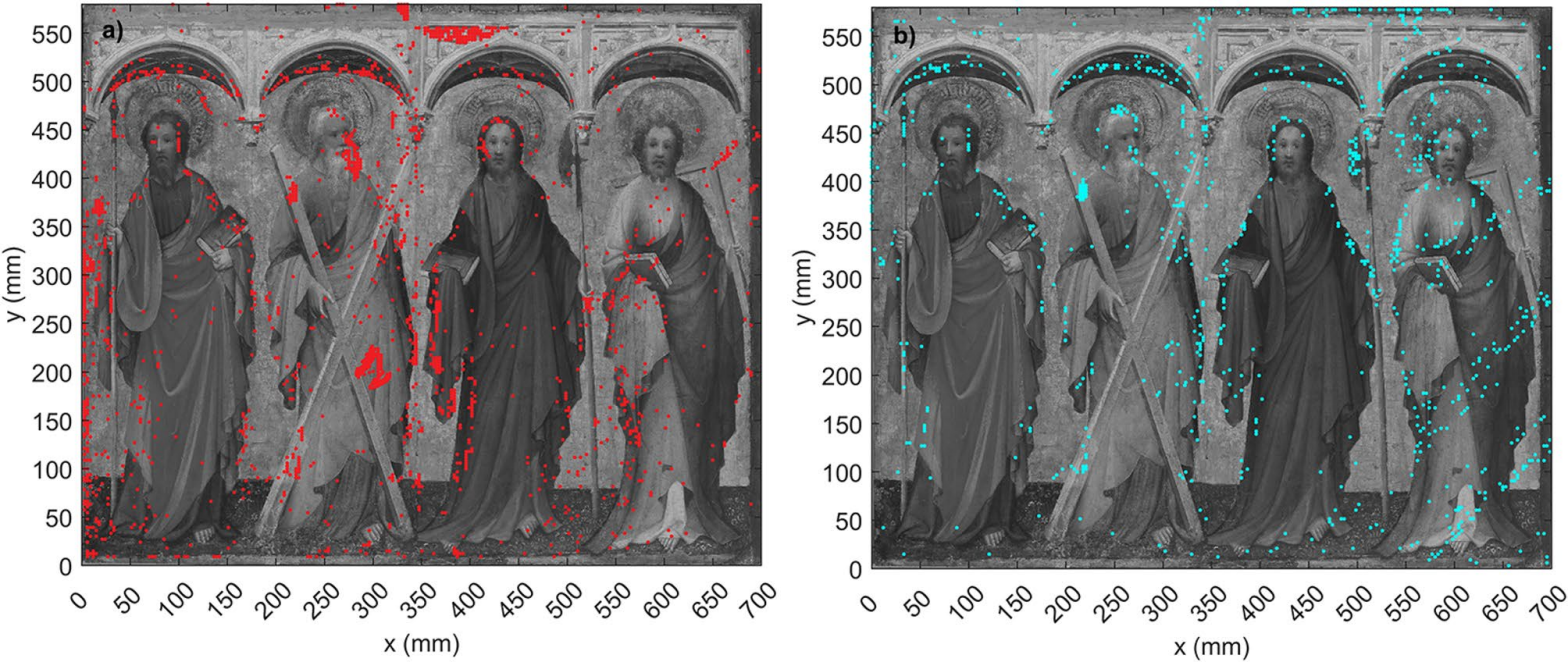
(**a**) Delamination mapping created with the THz data from before the consolidation effort plotted on top of a black and white photograph of the painting. (**b**) Delamination mapping after consolidation. Figure (**b**) shows significantly less areas that contain dense groups of markers, indicating the success of the consolidation intervention.

Figure 2 shows the mappings created with the algorithm from the before and after data separately, plotted on top of visible photographs of the painting. Figure 2a corresponds to the before data, and Fig. 2b shows the after data. Here we can more clearly see the success of the consolidation effort. We can again identify ROIs 2, 4 and 5. While these three regions contain densely positioned points on Fig. 2a, the same regions on Fig. 2b are for the most part free of points, with the exception of a few markers near Andrew’s mouth and cross.

**Figure 3:**
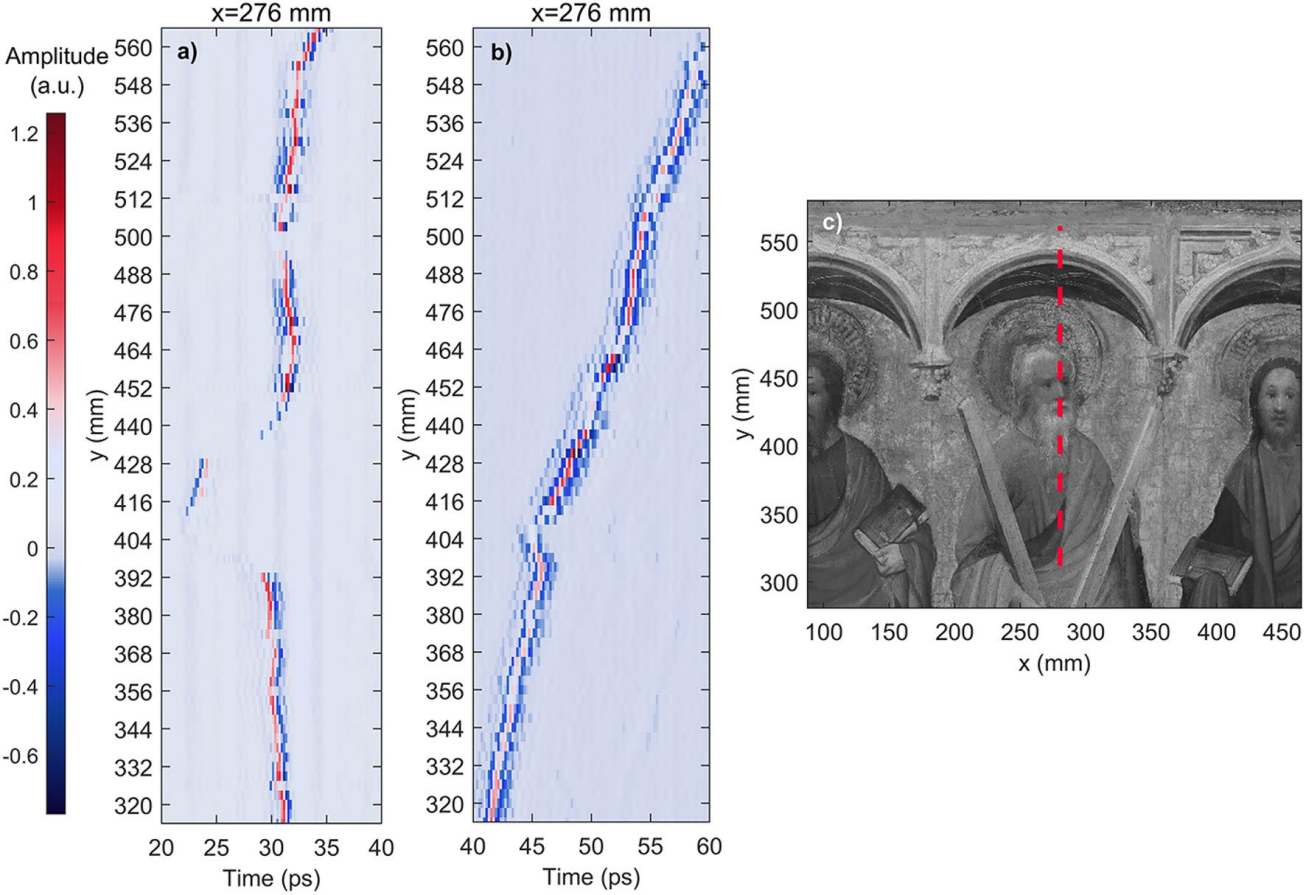
Comparison of before and after B-scans of ROI 2 at x = 276 mm. (**a**) B-scan along y-axis of the before data. Significant layer detachment is visible from y = 386 mm to y = 446 mm. Sub-surface air gaps are visible from y = 452 mm to y = 464 mm. (**b**) B-scan corresponding to the same region after the consolidation. The detached area between y = 386 mm and y = 446 mm has been consolidated, and appears homogeneous. Air voids are still present in the upper region along this line, as they were not consolidated. (**c**) Close up photograph of the region analyzed. The red dotted line shows the exact location on the x-axis where the data for the B-scans was obtained.

**Figure 4:**
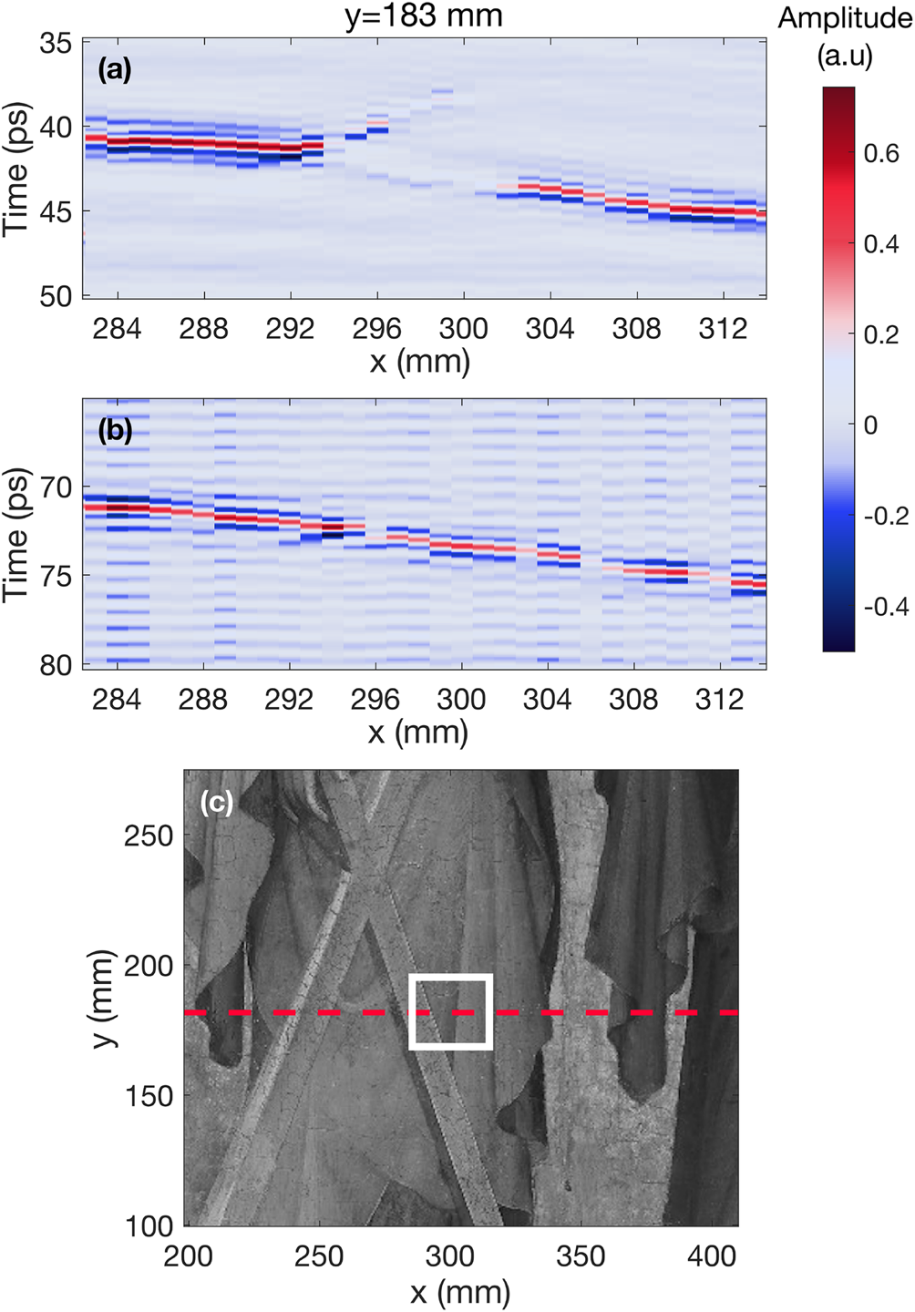
Before and after B-scans along x-axis of ROI 5. (**a**) B-scan taken from before data at y = 183 mm. The lifting of the outer paint from the inner paint layers is visible from x = 294 mm to x = 302 mm. (**b**) B-scan of the same region taken from the THz data after the consolidation. The same region that had become detached now appears homogeneous. (**c**) Close up of the region on the painting where B-scans were taken. The red dotted line indicates the exact location on the y-axis where the data was obtained, and the white rectangle encloses the area shown in the B-scans.

Two of the regions of interest from Fig. 1 were selected for more in depth analysis, as shown in Figs.  3 and 4. Figure 3 is one of the regions of interest where particularly severe delamination was detected: the area by Andrew’s head. Here, several types of irregularities were found: lifting, layer detachment and air gaps. Figure 3a shows a B-scan along the y-axis taken from the data before the consolidation. A clear separation can be seen from y = 386 mm to y = 446 mm, as well as several other layer irregularities such as air voids, located between y = 452 mm and y = 464 mm. Figure 3b shows a B-scan created from the data taken after the conservation intervention. It is clear that the consolidation effort on this area was successful, as only some smaller irregularities can be observed within the layers, but the area where the large piece was detached has been consolidated. The data for both B-scans was obtained at the same point on the x-axis (x = 276 mm), denoted by a red dotted line in Fig. 3c.

Figure 4 corresponds to the analysis of the ROI located on Andrew’s cross (region 5) that was identified by conservators to be a particularly problematic area. This region had a large air gap, noticeable in raking light. It was scanned with THz with a smaller step size (1 mm) in order to obtain more information regarding the internal structure. Figure 4a shows a B-scan taken along the x-axis of the before data from this area. In (a) it is clear to see that the paint layers are lifting from the support from x = 294 mm to x = 302 mm. Figure 4b shows a B-scan from the same region after the consolidation. In (b) the defect almost completely disappeared throughout the area in question, indicating that the consolidation was successful. Figure 4c shows a close up photograph of the region where the data for the B-scan was taken on Andrew’s cross. The red dotted line indicates the fixed position on the y-axis where the data for both B-scans was obtained, and the white box encloses the area along the x-axis that was analyzed.

## Discussion

The results obtained from the before and after terahertz data were compared with the results from the independent analysis of the conservators. Many of the regions of interest identified through THz were consistent with those identified by other means. However, several regions that had not been initially identified by conservators became apparent in the THz results. Such is the case of the area located on Philip’s spear (ROI 1), as well as the area on the upper left side of Andrew’s cross (ROI 3). In both of these regions dense groups of red and blue dots are present, and overlap each other almost exactly. It is possible that due to the type of delamination present on these areas, they were not identified in the initial analysis of the conservators.

Furthermore, using our algorithm was far more efficient than other means (i.e., analyzing individual B-scans) for identifying and mapping the regions of interest. In this case, some analysis of the B-scans was still required to corroborate the results, as well as to obtain more information regarding the type of layer separation present at different spots. The algorithm was able to identify with equal success all different types of irregularities including layer lifting and air voids. Determining the parameters used in the algorithm also required time and in depth analysis of the data in order to pinpoint only areas that presented abnormalities in their internal structure. In some areas, it was more difficult to apply the algorithm due to the surface angle of the painting. In places where the layer lifting or air gap was particularly severe, the angle between the THz emitter and the sample surface did not remain constant, resulting in noise and loss of signal at some points, which were not identified by the algorithm.

The mapping created using the algorithm was useful to determine the areas that required individual analysis. Through the observation of the before and after B-scans of these areas it was determined that the consolidation intervention was successful. It was also possible to determine the exact locations of smaller irregularities still present on the painting that were not consolidated. This type of analysis and images will be particularly useful to conservators in future conservation interventions, in an effort to avoid recurrent delamination.

## Conclusions

From the results we conclude that THz-TDI is a valuable tool for aiding consolidation interventions on panel paintings. In comparison with other methods used for this type of monitoring, it provides information regarding the internal structure of the sample in a completely non-destructive way. Due to its unique characteristics, this type of non-ionizing radiation can provide information about the complete stratigraphy of the object, from the surface to the wooden support, while other technologies are limited to the top layers. Furthermore, THz can be used to image entire artworks in an efficient manner. The portability of THz systems is a another advantage, as often the access to artworks is very restricted depending on the condition of the object. Therefore, it is highly advantageous that diagnostic tools in this field be quick, efficient and not limited by resources or space for their operation.

B-scans have repeatedly been found to be useful when using THz as a diagnostic tool for art conservation. However, they have not been as widely used for monitoring consolidation efforts. In this study, we have shown their value for determining types of layer separation present on a painting, and for evaluating the success of a consolidation intervention. Analysis of the cross-sectional images provided more information regarding the type of delamination present at different points. From these images it was possible to differentiate layer separation from lifting from sub-surface air gaps. This information is also very useful for conservators, as it aids their decisions regarding which regions to consolidate or not. B-scans, along with delamination mappings, can be useful for conservators in the future to aid their conservation efforts and recognize areas of interest that might not have been recognized using other methods.

## Methods

### Instrument and protocol

For this study, a Terawave (HHI/Toptica) terahertz spectrometer was used. The system consists of a 1.5 $\upmu$m femtosecond fiber laser (Toptica Femto FErb 1560) coupled to InGaAs-based photoconductive antennas that serve as the emitter and detector. A transceiver module formed by the photoconductive devices and a THz-beam splitter as well as two TPX lenses, one to collimate the beam and one to focus the radiation on the sample, producing a focal diameter of the terahertz beam between $\sim$300 $\upmu$m-1 mm for the frequency range used. These two lenses also do the reverse process to the reflected radiation. The entire tranceiver module was mounted onto a motorized x,y-motion unit that allowed for scanning the sample. The terahertz data was obtained in reflection geometry every 3 mm with a time window of 120 picoseconds and an average of 20 waveforms for the before data. Each reflected waveform from the before data contained a total of 2400 points.

### Terahertz imaging

**Figure 5:**
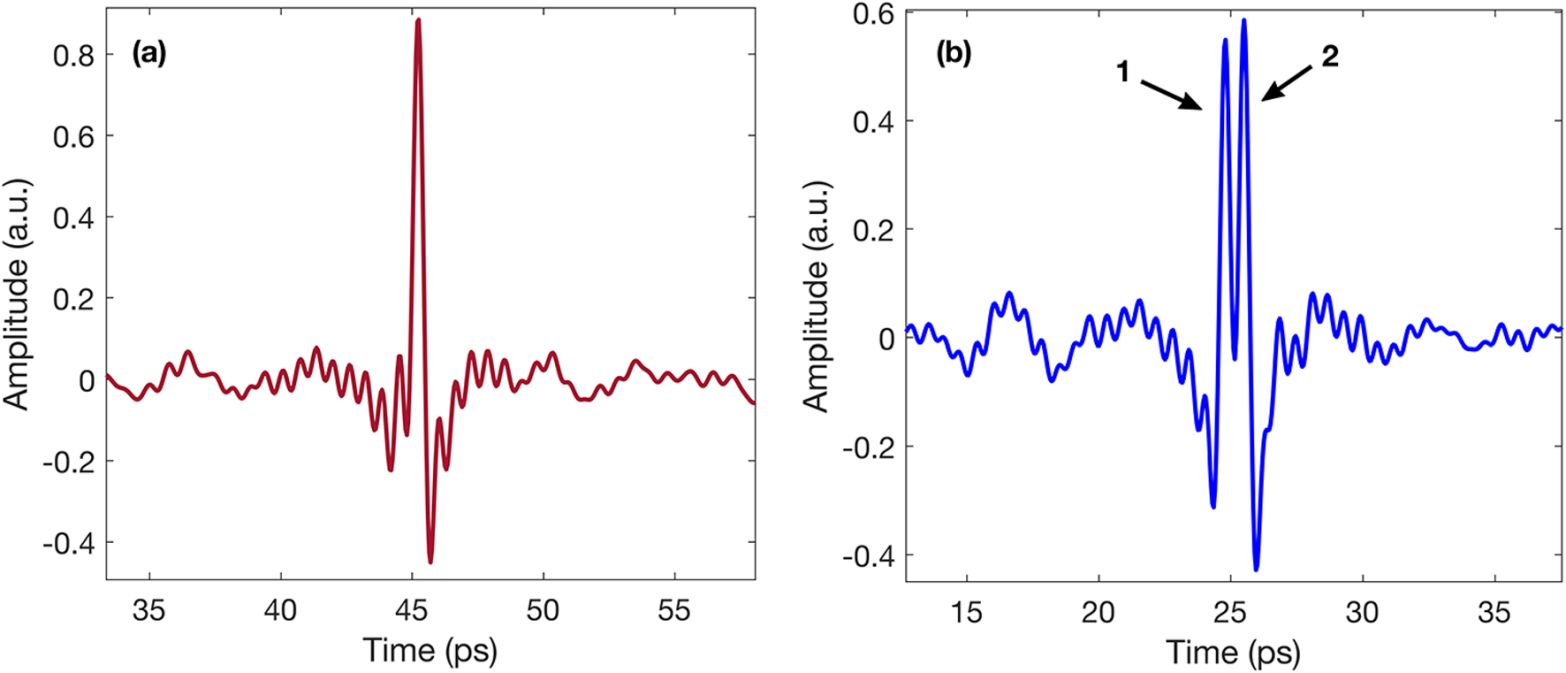
Comparison of regular and irregular waveforms for the mapping algorithm. (**a**) Shows a regular waveform corresponding to an area on the painting where the layers were homogeneous and no irregularities were present. (**b**) Shows an example of an irregular waveform, where layer detachment is denoted by the two peaks located between 20–30 ps. As peak 2 has an amplitude greater than 80% of peak 1, the algorithm saved the coordinates of this waveform.

Terahertz systems detect the electric field amplitude as a function of time at each specific point on a sample. The plot of this information is known as an A-scan. Figure 5 shows two examples of A-scans from different regions on the artwork. The initial peak, typically with the greatest amplitude, corresponds to the reflection from the air-paint interface at the surface, while the subsequent peaks are created by changes in refractive indices at each interface within the internal structure of the sample, which indicate the presence of multiple layers. It is possible to determine the thicknesses and depths of specific layers on a sample by measuring the optical distance between peaks shown on an A-scan^29^. A stratigraphic image (B-scan) can be reconstructed by accumulating a series of A-scans along a line of the sample, and assigning a color contrast mechanism to the peak amplitudes, as shown in Figs. 3 and 4. B-scans allow for a more intuitive analysis of terahertz data, as air voids and layer detachment can be easily identified in these images. In the figures presented in this research, the peaks with the greatest electric field amplitudes are assigned the colors bright red, for positive amplitude, and bright blue for negative amplitude. Air gaps can be identified as significant areas where the amplitude is close to zero (denoted by light purple) between interfaces with a clear positive or negative amplitude. Similarly, it is possible to identify layer detachments in these images by locating areas where two pulses generated in the neighborhood of the air gap are visible, but the amplitude in between is zero.

### Mapping algorithm

In order to create the image displayed in Fig. 1a, an algorithm was developed using MATLAB. Through individual analysis of A-scans and B-scans from the regions of interest, it was possible to determine the criteria that were implemented in the algorithm to differentiate between areas that presented layer irregularities, and areas that appeared to be in good condition.

Figure 5a shows an example of an A-scan from a “regular” region, or area where the layer distribution was homogeneous. We observed that the A-scans from “irregular” areas, that is, areas where there were clear layer separations or air gaps, contained a large peak, followed by one or more other peaks with similar amplitudes, as shown in Fig. 5b.

It was determined that if the second peak in the A-scan had an amplitude of at least 80% of the first peak, the coordinates of this point should be saved. The regions identified by the algorithm using this condition were correlated with regions where delamination had been previously identified by conservators, in order to ensure the accuracy of the results.

The 80% condition was implemented into the algorithm, which scanned all of the A-scans and stored the indices of the data that complied with this criteria. This was done for the complete set of data before and after the consolidation. The coordinates of the irregular points were then plotted on top of a visible photograph of the painting, thus providing a map of the areas in need of consolidation as shown in Fig. 1b. The areas identified by the algorithm were corroborated by visual analysis of the B-scans in order to confirm that the 80% condition produced the most accurate results.

### Conservation process

Before the treatment, the painting was analyzed by conservators independently from the THz measurements. A detailed examination of the areas to be consolidated was done by using visual observation in raking light and under the microscope. As a result, two different types of detachment were found. There were detachments of the paint layer from the textile lamination, and blisters due to the detachment of this textile from the wooden panel, even in places where the paint layer was still well adhered to the textile. These damages, which have been recurring over the past decades, cannot be attributed only to aging or storage factors such as climate, but are possibly inherent in the production process of this specific painting. After weighing the possible risks, it was decided that areas that were stable, or where there was a risk of breaking or compressing the paint layers by laying them down, should not be consolidated.

3% and 5% Sturgeon’s Glue was used as a consolidating agent. Partly to ensure better penetration, pre-wetting was used. The areas could then be laid down with the heating spatula under low heat and light pressure. Currently, the painting is monitored regularly so that early intervention can be made if new changes occur.

## Data Availability

The datasets used and/or analysed during the current study available from the corresponding author on reasonable request.
